# Screening Plant Growth-Promoting Bacteria with Antimicrobial Properties for Upland Rice

**DOI:** 10.4014/jmb.2402.02008

**Published:** 2024-04-01

**Authors:** Khammool Khamsuk, Bernard Dell, Wasu Pathom-aree, Wanwarang Pathaichindachote, Nungruthai Suphrom, Nareeluk Nakaew, Juangjun Jumpathong

**Affiliations:** 1 Department of Agricultural Science, Faculty of Agriculture, Natural Resources and Environment, Naresuan University, Phitsanulok 65000, Thailand; 2 Centre for Crop and Food Innovation, Murdoch University, 90 South St., Murdoch WA, 6150 Australia; 3 Department of Biology, Faculty of Science, Chiang Mai University, Chiang Mai, 50200, Thailand; 4 Center of Excellence in Research for Agricultural Biotechnology, Naresuan University, Phitsanulok 65000, Thailand; 5 Center of Excellence in Biomaterials, Naresuan University, Phitsanulok 65000, Thailand; 6 Department of Chemistry, Faculty of Science and Center of Excellence for Innovation in Chemistry, Naresuan University, Phitsanulok 65000, Thailand; 7 Department of Microbiology and Parasitology, Faculty of Medical Science, Naresuan University, Phitsanulok 65000, Thailand; 8 Centre of Excellence in Fungal Research, Naresuan University, Phitsanulok 65000, Thailand

**Keywords:** Lipopeptides, surfactin, TBRC 15998, root-associated bacteria

## Abstract

This study explores beneficial bacteria isolated from the roots and rhizosphere soil of Khao Rai Leum Pua Phetchabun rice plants. A total of 315 bacterial isolates (KK001 to KK315) were obtained. Plant growth-promoting traits (phosphate solubilization and indole-3-acetic acid (IAA) production), and antimicrobial activity against three rice pathogens (*Curvularia lunata* NUF001, *Bipolaris oryzae* 2464, and *Xanthomonas oryzae* pv. *oryzae*) were assessed. KK074 was the most prolific in IAA production, generating 362.6 ± 28.0 μg/ml, and KK007 excelled in tricalcium phosphate solubilization, achieving 714.2 ± 12.1 μg/ml. In antimicrobial assays using the dual culture method, KK024 and KK281 exhibited strong inhibitory activity against *C. lunata*, and KK269 was particularly effective against *B. oryzae*. In the evaluation of antimicrobial metabolite production, KK281 and KK288 exhibited strong antifungal activities in cell-free supernatants. Given the superior performance of KK281, taxonomically identified as *Bacillus* sp. KK281, it was investigated further. Lipopeptide extracts from KK281 had significant antimicrobial activity against *C. lunata* and a minimum inhibitory concentration (MIC) of 3.1 mg/ml against *X. oryzae* pv. *oryzae*. LC-ESI-MS/MS analysis revealed the presence of surfactin in the lipopeptide extract. The crude extract was non-cytotoxic to the L-929 cell line at tested concentrations. In conclusion, the in vitro plant growth-promoting and disease-controlling attributes of *Bacillus* sp. KK281 make it a strong candidate for field evaluation to boost plant growth and manage disease in upland rice.

## Introduction

Upland rice (*Oryza sativa* L.) is predominantly cultivated in the mountainous terrains of Asia, Africa, and South America [1]. Numerous indigenous varieties of upland rice are grown as traditional crops on sloped and hilly terrains under rainfed conditions in Thailand [2]. Although these varieties possess an inherent capacity for adaptability to challenging environmental conditions, they typically have lower yields compared to their lowland counterparts. The application of inorganic fertilizers has been shown to increase rice yield but at the expense of soil quality degradation [3]. In the context of sustainable agriculture, the deployment of effective microbial agents as biofertilizers in upland rice cultivation could offer a viable strategy for soil health improvement while mitigating environmental impact.

The rhizosphere, comprising the endorhizosphere, rhizoplane, and ectorhizosphere, plays a critical role in shaping soil microbial communities [4]. The rhizosphere serves as a hotspot for microbial activity depending on the availability of root exudates [5]. The rhizosphere can harbor a diversity of bacteria, fungi and nematodes depending on factors such as the soil type, rice variety, rhizodeposition and environmental conditions [6, 7]. For rice rhizospheres, bacterial populations can be isolated using traditional serial dilution techniques [8]. Advances in high-throughput sequencing technology have further facilitated investigations into the structure and diversity of these bacterial communities [9]. In a comprehensive review by Vessey [10], multiple bacterial genera including *Bacillus*, *Enterobacter*, and *Pseudomonas* were identified from various rhizospheres and recognized as plant growth-promoting rhizobacteria (PGPR). Additionally, Hallmann *et al*. [11] reported that *Bacillus*, *Enterobacter*, *Pseudomonas*, and *Agrobacterium* constituted the predominant bacterial endophytes isolated from plant tissues.

The PGPR exhibit both direct and indirect positive impacts on plants and soil through the synthesis of growth-promoting compounds, including indole-3-acetic acid and siderophores [12]. Additionally, it has been observed that bacteria secrete 1-aminocyclopropane-1-carboxylate (ACC) deaminase [13] and other organic substances in order to facilitate the dissolution of phosphate in soil [14]. The release of antagonistic agents and antibacterial chemicals can indirectly benefit crop yields by reducing damage from plant pathogens [15]. In particular, bacterial lipopeptides are pivotal in the control of some plant pathogens. For example, the surfactin, fengycin and iturin lipopeptides from *Bacillus* are active against a wide range of plant pathogens [16, 17]. Among these, surfactin has been implicated in the biological control of the plant pathogens *Curvularia lunata*, *Fusarium semitectum*, *Helminthosporium oryzae* [18], and *Xanthomonas oryzae* pv. *oryzae* (*Xoo*) [19]. Thus, lipopeptides continue to be explored for their benefits in sustainable agriculture and pest management [20, 21].

Most studies on rice-associated bacteria have been undertaken with paddy or lowland rice and there is only limited investigation into bacteria associated with rain-fed upland rice [22, 23]. As environmental conditions can differ greatly between upland regions engaged in rain-fed rice production, there is a need to target beneficial organisms to local conditions. Therefore, in this study we isolated bacteria from root tissues and rhizosphere soils from upland rice, and screened strains for their ability to solubilize insoluble calcium phosphate and produce IAA, and their antimicrobial activity against rice pathogens in vitro. In addition, crude lipopeptides produced by *Bacillus* sp. KK281 were isolated and characterized using liquid chromatography–electrospray ionization–tandem mass spectrometry (LC–ESI–MS/MS), and crude extracts were assessed for antimicrobial activity and cytotoxicity. The overall objective was to discover bacterial strains with potential for improving the growth and sustainable yield of upland rice in Thailand.

## Materials and Methods

### Isolation of Rice Root-Associated Bacteria

Ten samples of Khao Rai Leum Pua Phetchabun rice plants at the panicle initiation stage of development were harvested from local farms in Khao Kho district, Phetchabun Province, Thailand. Root samples were soaked in phosphate-buffered saline (PBS) and run through a sonicator (CREST Ultrasonics, USA) for 30 sec to obtain rhizosphere bacteria via serial ten-fold dilutions. For obtaining endophytic bacteria, 2-3 cm pieces of roots were surface sterilized as described previously [24, 25]. The sterilized roots (ca. 1 g) were mixed with 1 ml of 0.03 M MgSO_4_ and ground in a mortar. Serial ten-fold dilution of ground roots was prepared in PBS to 10^-3^-10^-7^. Aliquots (100 μl) of root suspension were spread onto Luria-Bertani agar (LBA), King's B agar (KB) tryptic soya agar (TSA) and peat moss extract agar. Plates were incubated in the dark for 5 days at 28-30°C and then colonies were observed under a stereomicroscope (Optika, Italy). Bacterial strains were characterized based on colony morphology, Gram staining, and cell morphology using a scanning electron microscope (SEM) (Leo1455VP). All bacterial cultures were kept at –80°C in a 25% (v/v) glycerol solution and used for further research. In vitro assays for measuring phosphate solubilization, indole-3-acetic acid production (IAA), and antimicrobial production were determined using all 315 isolates (127 endophytes and 188 from the rhizosphere).

### Screening for Phosphate Solubilization Activity

Bacterial strains were tested for phosphate solubilizing activity on Pikovskaya's medium (PVK) containing 0.5% (w/v) tricalcium phosphate (Ca_3_(PO_4_)_2_). Bacteria were aseptically spot inoculated on agar plates and incubated at 28°C ± 2°C for 3-7 days. Later, the diameter of the clear zone was measured (in mm) using a Vernier caliper, and the measurements used for a phosphate solubilization index (PSI) [26]. The released phosphorus was quantified using the molybdate blue/colorimetric method [27]. The ninety-one isolates that were able to solubilize insoluble calcium phosphate on PVK were investigated further. Bacterial inoculum (1 × 10^8^ CFU/ml) was inoculated into 250 ml Erlenmeyer flasks containing 50 ml Pikovskaya's broth supplemented with 0.5% (w/v) tricalcium phosphate and incubated in a 30°C rotary shaker at 150 rpm, for 7 days. After incubation, the supernatant was collected by centrifugation (Dynamica, Velocity14 R) at 10,000 ×*g* for 10 min at 4°C, and the pH was measured with a pH meter (Sartorius, PB-10).

### Screening for Indole-3-Acetic Acid Production

The ability of all 315 isolates to produce IAA was assessed as described by Gordon & Weber [28]. Bacterial cells were cultivated in Yeast malt broth (YMB) containing 1 mg/ml L-tryptophan and shaken at 150 rpm for 4 days at room temperature (28-30°C). The supernatant was collected by centrifugation (Dynamica, Velocity14 R) at 10,000 ×*g* for 10 min at 4°C. One ml of supernatant was mixed with two ml of Salkowski's reagent and incubated for 30 min in the dark, following the protocol by Sev *et al*. [29]. The absorbance of this mixture was measured at 530 nm using a spectrophotometer (Shimadzu, UV-1800) [30]. A standard curve was produced using analytical-grade IAA [31].

### Screening for Antagonistic Bacteria Using Dual Culture Bioassay

The dual culture method was used to evaluate the antifungal activity of 43 bacterial isolates (24 from the rhizosphere and 19 from within the roots). We selected 13 isolates (KK005, KK039, KK047, KK053, KK065, KK066, KK074, KK075, KK077, KK081, KK102, KK245 and KK291) with strong ability to produce IAA, 16 isolates (KK002, KK007, KK018, KK079, KK108, KK135, KK146, KK151, KK180, KK191, KK199, KK202, KK225, KK306, KK313 and KK314) with strong ability to solubilize phosphate, and a further 14 isolates (KK024, KK067, KK073, KK145, KK149, KK157, KK184, KK231, KK232, KK243, KK269, KK275, KK281 and KK287) with different colony morphologies from the original 315 isolates. The 43 isolates were used in dual culture bioassays against two pathogenic fungi of rice: *Curvularia lunata* NUF001 isolated from Khao Rai Leum Pua Phetchabun rice by the Agricultural Microbiology Laboratory, Faculty of Agriculture, Natural Resources and Environment, Naresuan University, and *Bipolaris oryzae* 2464 isolated from lowland rice which was provided by the Plant Protection Research and Development Office, Department of Agriculture, Thailand. The pathogens were cultured on Potato dextrose agar (PDA). A 5 mm plug of 5-day-old culture of each fungal pathogen was placed 2.5 cm away from the edge in a 90 mm diameter Petri dish containing PDA and incubated at 28°C ± 2°C for 1 day. Inoculant bacteria were grown on TSA for 24-48 h and then streaked 4.5 cm away from the plug of the pathogenic fungus on the same Petri dish and incubated at 28°C ± 2°C for nine days [18]. The percentage of inhibition of radial growth (PIRG) was calculated using the following formula:

PIRG (%) = [(R1 – R2)/R1] × 100

where R1 is the radial diameter of the control colony, and R2 is the radial diameter of the treatment colony.

### Assessment of Antifungal Activity Using Cell-Free Supernatant

Nine bacterial isolates, three (KK024, KK275 and KK281) with the highest fungal activity in the dual culture assay, and six (KK023, KK024, KK058, KK275, KK281 and KK312) with different colony morphology, were used for the cell-free supernatant assay. Inocula (ca. 10^6^ CFU/ml) were cultured in 5 ml of modified Wickerhams Antibiotic Test Medium (WATM) [32] and incubated at 28°C on a shaking incubator for 5 days. The cell-free supernatants were collected by centrifugation (Dynamica, Velocity14 R) at 15,000 ×*g* for 20 min at 4°C and filtered through a 0.22 μm membrane filter (Sartorius, Germany). Fungal spore suspensions were prepared from 3-day-old cultures on PDA plates, and adjusted to 1 × 10^5^ cells/ml by dilution with sterile distilled water using a hemocytometer (HBG, Germany). The agar well diffusion method was used to determine the antifungal activity of the cell-free supernatant [33]. Four wells of 5 mm diameter were punctured around the fungus. Aliquots (25 μl) of cell-free supernatant were added in each well. Prochloraz (500 ppm) (ZAGRO, Thailand) fungicide was used as a control. Plates were incubated at 28°C for 48 h. Antifungal activity was observed by measuring the diameter (mm) of the detectable zone of inhibition.

### Molecular Identification of Bacterial Strains

Three rhizosphere isolates (KK138, KK269 and KK281) and three endophytes (KK007, KK018 and KK066) were chosen for identification based on their overall ability to solubilize insoluble calcium phosphate, produce IAA, and their antimicrobial activity. The 16S rRNA gene was amplified with the 27F primer (5'-AGA GTT TGA TCM TGG CTC AG-3') and the 1492R primer (5'-TAC GGY TAC CTT GTT ACG ACT T-3') [34]. An amplification reaction was performed as previously described [35], and the purified PCR products were commercially sequenced at Macrogen, Korea. The obtained 16S rRNA gene sequences were compared to those of their phylogenetic relatives in the EzBioCloud database (http://www.ezbiocloud.net). The MEGA software package (Version 10) was used to construct and analyze phylogenetic trees using a neighbor-joining method [36]. The resultant tree topology was evaluated using bootstrap analysis with 1,000 resampled data sets. The sequences were submitted to the GenBank database to obtain accession numbers.

### Extraction of Lipopeptides

Isolate KK281 was chosen to investigate antimicrobial lipopeptides due to its high antimicrobial activity in the dual culture study and its antifungal activity in the cell-free supernatant test. Crude extract containing lipopeptides was prepared using a slightly modified method from that described by Pathak *et al*. [37]. KK281 was grown in Minimum salt liquid medium (MSLM) [16] at 28 ± 2°C for 72 h; then the culture broth was centrifuged at 15,000 ×*g* for 20 min at 4°C. Lipopeptides were precipitated by adding 6.0 N HCl to a final pH of 2 and stored overnight at 4°C. The precipitate was collected using centrifugation (Dynamica, Velocity14 R) at 15,000 ×*g* for 20 min at 4°C, washed twice with sterile water, extracted using anhydrous methanol, and filtered with 0.22 μm PES membrane filter (Johnson Test Papers) to remove cell debris or larger particles. The combined extracts were evaporated at 45°C under vacuum to dryness in a rotary evaporator and were lyophilized to yield a pale-yellow extract of lipopeptides.

### LC–ESI–MS/MS Conditions

The crude lipopeptide extract was dissolved in 70% ethanol (10 mg/ml) and standard surfactin was prepared in ethanol (5 mg/ml), sonicated and filtered using a syringe filter (0.45 μm) and the analysis parameters as described by Ma *et al*. [38]. A Phenomenex Luna C-18 (2) column (5 μm, 150 × 4.6 mm) thermostabilized at 35°C was used for chromatographic separation. A 10 μl sample was injected into the LC system and eluted with a combination of water (solvent A), and acetonitrile (solvent B). Both solvents contained 0.1% (v/v) formic acid for facilitating the protonation of the nitrogen compounds, yielding protonated molecules [M + H]^+^. The gradient elution with a 0.5 ml/min flow rate was: 0 min (10% B), 0-5 min (10-50% B), 5-7 min (50-95% B), and 7-30 min (held at 95% B). The post run was set for 5 min before a new injection. The ESI-MS analysis was carried out in a positive mode with a mass range from *m/z* 100 to 1700 amu and the following operating conditions: capillary voltage +3,500 V; drying gas (N_2_) 10 L/min; dry gas temperature at 350 °C; and nebulizer pressure 30 psig. The fragmentations were performed using automatic MS/MS experiments with collision energies fixed at 10, 20, and 40 V. Compounds were identified by comparing their retention time, MS data, MS/MS fragmentation profiles to those of standard compounds, the chemistry database ChemSpider and by comparison with MS data previously reported in the literature [39].

### Antifungal Activity of Lipopeptides

The antifungal activity of the extracted lipopeptides was evaluated by the disc diffusion assay against *C. lunata* NUF001 using lipopeptide crude extracts that were dissolved in 70% ethanol at three concentrations: 125, 250 and 500 ppm. The fungal spore suspension was prepared and adjusted to 1 × 10^5^ cells/ml in sterile distilled water using a hemocytometer (HBG). Filter paper discs were impregnated with lipopeptide extract and placed on a PDA plate. A disc impregnated with Prochloraz (500 ppm) and a disc impregnated with 70% ethanol were used as controls. The plates were incubated at 28 ± 2°C for 48 h, and the diameters (mm) of inhibition zones were recorded.

### Antibacterial Activity of Lipopeptides

The antibacterial activity of the lipopeptides was evaluated by the disc diffusion assay by preparing a 25,000 ppm stock and dissolving lyophilized fractions in 70% ethanol at three concentrations: 62.5, 125, 250, and 500 ppm. A paper disc (6 mm diameter) containing lipopeptides was placed on Nutrient agar (NA) inoculated with *Xanthomonas oryzae* pv. *oryzae* (*Xoo*). A streptomycin (10 μg/disc) and chloramphenicol (10 μg/disc) impregnated disc was used as a control. The plates were incubated at 28 ± 2°C for 72-96 h and the inhibition zones (mm) were measured (included the paper disc diameter).

### Cytotoxicity Testing

Cytotoxicity testing was undertaken on the L-929 cell line [40] to establish whether the bacteria and extracts would be safe to use in future in vivo screening trials. The standard colorimetric MTT (3-[4,5-dimethylthiazol-2-yl]-2,5-diphenyltetrazolium bromide) assay with the L-929 cell line (mouse fibroblast) was performed. The crude extracts of KK281 were prepared in a 5-80 μg/ml concentration range and were dissolved in 100 μl DMSO. A microplate spectrophotometer (Shimadzu, UV-1800) was used to measure absorbance at 570 and 650 nm. Doxorubicin was used as the positive control. Cytotoxicity was expressed as the concentration of the compound inhibiting growth by 50% (IC_50_).

### Statistical Analysis

All experiments were carried out in triplicate except the LC-ESI- MS/MS analysis. The data were subject to analysis of variance (ANOVA) followed by Duncan’s multiple range test (*p* < 0.05) using SPSS Statistics 17.0 for Windows (Trial version).

## Results

### Isolation of Rhizosphere and Endophytic Bacteria

**Fig. 1:**
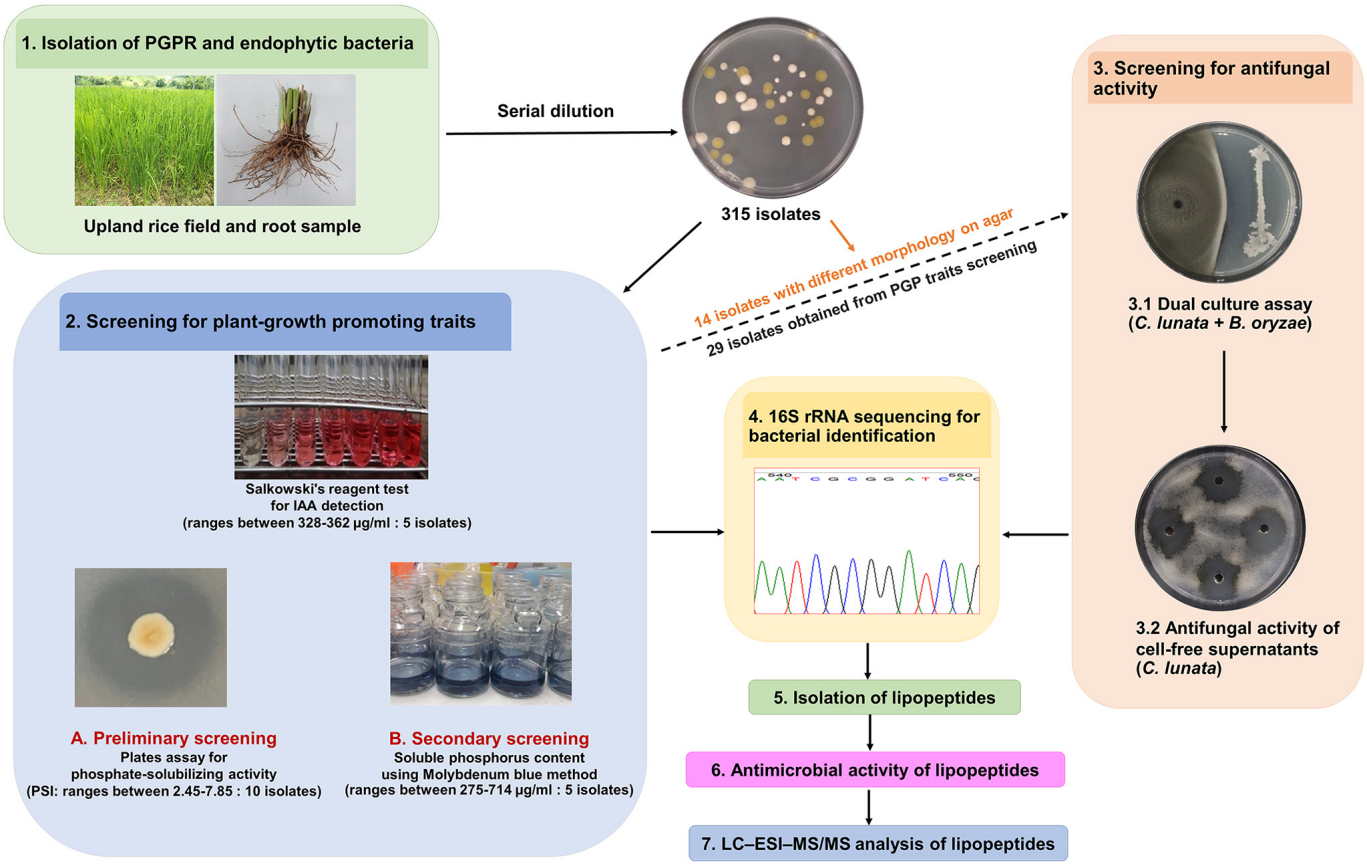
Schematic diagram illustrating the steps required for isolating and screening for plant growthpromoting bacteria and assessing their potential antibacterial properties.

A total of 315 culturable bacterial isolates were obtained, 127 from root tissues and 188 from the rhizosphere of upland rice. Ninety isolates were recovered from Luria-Bertani agar, of which 31 were endophytic bacteria and 59 were rhizospheric bacteria. Fifty of the 82 isolates from King's B agar were rhizosphere bacteria, and the remainder were endophytes. Furthermore, 81 isolates were obtained using Tryptic soya agar (38 rhizospheric, 43 endophytic) and 62 isolates (41 rhizospheric, 21 endophytic) were obtained from the peat moss extract agar. All isolates were investigated in vitro for phosphate solubilizing activity and IAA production. Selected isolates were used subsequently for antifungal, lipopeptide and other studies (Fig. 1).

### Indole-3-Acetic Acid Production

Out of 315 isolates, five isolates were able to produce IAA in the range of 328-362 μg/ml (Table 1), with isolate KK074 the highest producer (362.6 ± 28.0 μg/ml).

### Phosphate Solubilization

In the primary screening, forty-six isolates were able to solubilize insoluble calcium phosphate on Pikovskaya’s plates (data not shown). Out of forty-six isolates, ten exhibited phosphate solubilization index ranged from 2.45 to 7.85, with KK225 having the highest PSI of 4.51 (3 days) and 7.85 (7 days) (Table 1). Five isolates were able to release phosphorus in the range of 275-714 μg/ml in the secondary screening with the molybdenum blue method. Isolate KK007 released the highest amount of phosphorus (714.25 ± 12.14 μg/ml).

### Screening of Antagonistic Bacteria by Dual Culture Bioassay

Of the 43 isolates investigated for their antifungal activities, six isolates inhibited *C. lunata* NUF001 more than 50%, and KK024 was the most potent antagonist (Table 1) with 58% inhibition (Fig. S1B). Among nine isolates, KK269 inhibited growth of *B. oryzae* 2464 by 56% (Fig. S1D). Overall, eleven isolates were found to inhibit at least one fungal pathogen (Table 1).

### Antifungal Activity of Cell-Free Supernatants

Cell-free supernatants of the nine bacterial isolates that were tested showed antifungal activity against *C. lunata* NUF001 (Fig. S2). Among these, KK281 and KK288 had the highest antifungal activity (21.15 ± 1.00 mm and 21.62 ± 0.55 mm), respectively (Table S1).

### Traits of Bacterial Isolates Selected for Further Study

**Fig. 2:**
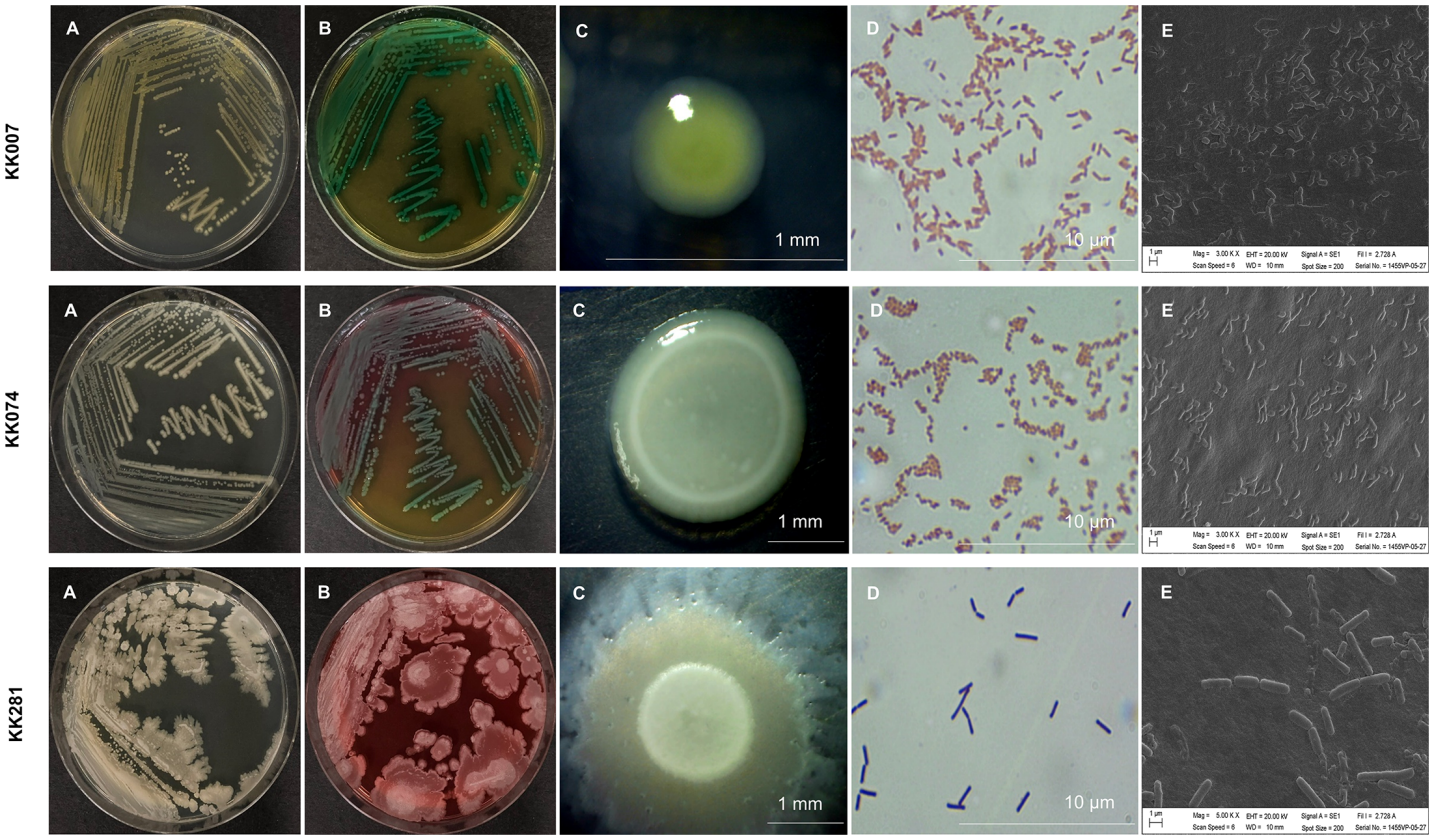
Isolation and characterization of bacterial isolates KK007, KK074 and KK281: (**A**) morphological characterization on TSA; (**B**) on HiCrome *Bacillus* Agar; (**C**) colony morphology; (**D**) Gram staining; (**E**) appearance under SEM.

Based on indole-3-acetic acid production, phosphate solubilization and antifungal assays, isolates KK007, KK074 and KK281 were selected for further assays. Colony morphology (Fig. 2C) was characterized on TSA (Fig. 2A) and HiCrome *Bacillus* Agar Base (Fig. 2B), followed by Gram staining (Fig. 2D) and scanning electron microscopic (SEM) analysis (Fig. 2E).

### Identification of Bacterial Isolates Based on 16S rRNA Gene and Phylogenetic Tree

Based on screening program of plant growth promoting properties, antimicrobial activity and colony morphology observation, we identified at least six genera using molecular analysis (Table 2). Among those isolates, KK281 was selected for further assays due to its superior bioactivities. BLAST analysis revealed that the 16S rRNA gene of isolate KK281 was 99.86% identical to that of *Bacillus* siamensis KCTC 13613^T^ (Table 2). Phylogenetic analysis (Fig. S3) showed that KK281 was clustered in the same clade with *B. siamensis* YC-9 (GenBank Accession No. MN166187). The 16S rRNA gene sequence of KK281 was submitted to GenBank under the accession number ON406151. Isolate KK281 was deposited at the Thailand Bioresource Center and assigned the number TBRC 15998.

### Antimicrobial Activity of Lipopeptides

**Fig. 3:**
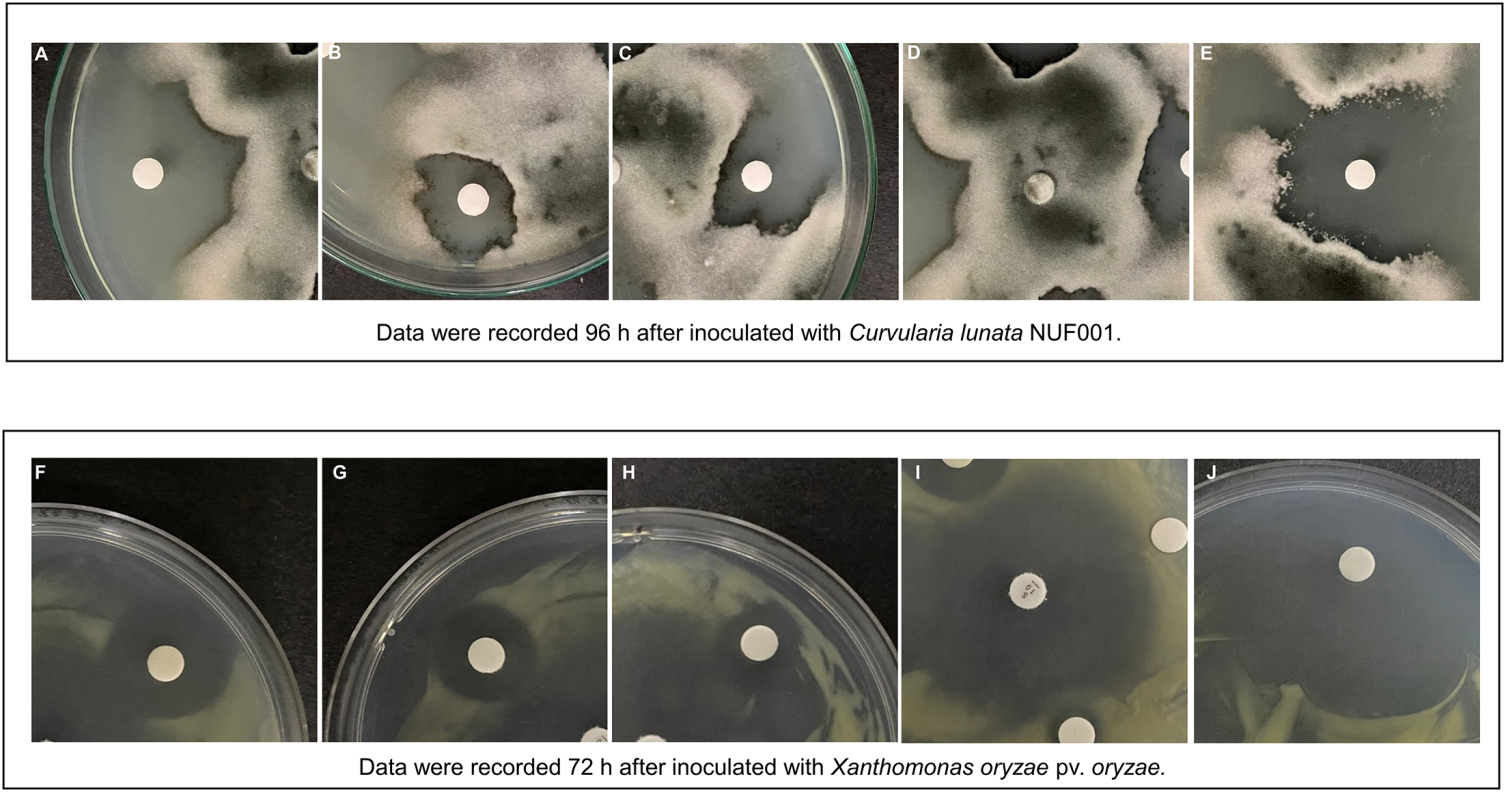
Inhibition zone diameter (mm) of lipopeptide extracts from isolate KK281 against fungal pathogens *Curvularia lunata* NUF001. (**A-E**) on PDA at 28°C ± 2 for 96 h) and *Xanthomonas oryzae* pv. *oryzae* (F-J) on NA plate at 28°C ± 2 for 72 h. (**A,F**) 12.5 mg/ml; (**B,G**) 6.25 mg/ml; (**C,H**) 3.125 mg/ml; (**D**) 70% ethanol; (**E**) Prochloraz 500 ppm; (**I**) streptomycin 10 μg/disc and (**J**) chloramphenicol 10 μg/disc.

**Antifungal Activity.** Antifungal activity of the lipopeptides are presented in Fig. 3. The crude lipopeptides produced by *Bacillus* sp. KK281 significantly inhibited the growth of *C. lunata* NUF001 and the strongest inhibitory activity of 39.93 ± 2.14 mm was observed at a crude extract concentration of 12.5 mg/ml (Table 3).

**Antibacterial Activity.** Crude lipopeptides at a concentration of 12.5 mg/ml resulted in a large zone of inhibition (19.33±1.29 mm) against *Xoo* in the agar diffusion assay (Table 3). The antifungal activity of lipopeptides showed a MIC value of 3.125 mg/ml after 72 h of growth.

### Liquid Chromatography–Electrospray Ionization–Tandem Mass Spectrometry (LC–ESI–MS/MS) Analysis of Lipopeptides

**Fig. 4:**
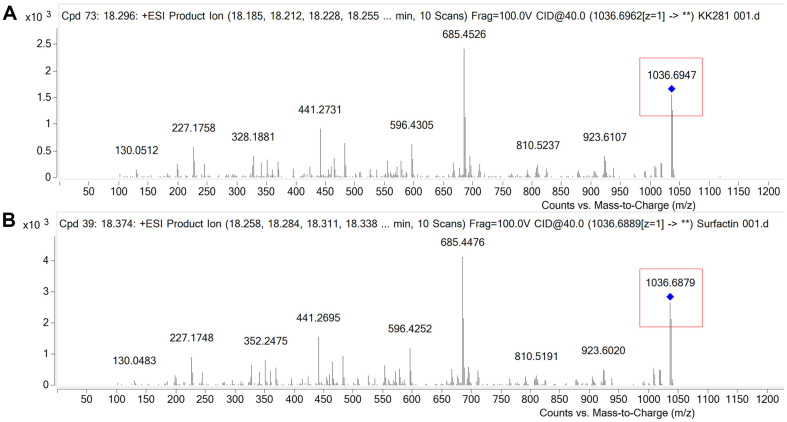
LC–ESI–MS/MS spectra of surfactin. (**A**) Crude lipopeptides of KK281 precursors ion [M + H]^+^at *m/z* 1036.6947 at retention time 18.296 min. (**B**) Standard surfactin precursors ion [M + H]^+^at *m/z* 1036.6879 at retention time 18.374 min.

The chemical profile of crude lipopeptide extract was investigated using LC-ESI-MS/MS instrument. The full scan total ion chromatogram of sample demonstrated that the main peaks began at 18 min. In the range of 18.179–18.557 min, the major peaks of surfactin were eluted at a retention time (*t*_R_) of 18.296 min. The MS/MS was operated in the selected target precursor ion *m/z* 1036 for surfactin. As shown in Fig. 4A, the major component consisted of *m/z* 1036.6947 [M + H]^+^ and presented MS/MS fragments corresponding to losses of amino acid residues, with *m/z* values of 923.6107, 810.5237, 685.4526, 596.4305, 441.2731, 328.1881, 227.1758 and 130.0512, respectively. In comparison to the standard (Fig. 4B), this compound was identified as surfactin (C_53_H_93_N_7_O_13_).

### Cytotoxicity Testing

Our results showed no cytotoxic effects of the crude lipopeptide derived from *Bacillus* sp. KK281 at concentrations ranging from 5 to 80 μg/ml in L-929 mouse fibroblast cells. By contrast, doxorubicin began to cause cytotoxicity at a concentration of 0.402 ± 0.040 μg/ml. The absence of cytotoxicity in the crude lipopeptide suggests its potential for further testing at the greenhouse stage.

## Discussion

In this study, we employed a combination of morphological observations and molecular techniques for identifying plant growth-promoting bacteria (PGPB). The 16S rRNA gene sequencing was used to identify and compare the taxonomical differences with those recorded in databases (GenBank and EzBioCloud Database). This approach allowed us to accurately categorize the isolates at the genus level, encompassing members of the genera *Bacillus*, *Enterobacter*, *Pseudomonas*, *Pantoea*, and *Acinetobacter*, which were identified as predominant taxa with PGPR properties in the root tissues and rhizosphere soils of upland rice. In comparison, *Bacillus* species were reported to be most abundant in root samples of Indian rice when using metagenomic analysis [41].

Phosphate solubilization is one of the most important plant growth-promoting traits due to the limited supply of available phosphate resulting from the strong adsorption of phosphate to reactive surfaces in most agricultural soils. The principal mechanism of phosphate solubilization by bacteria is the production of organic acids and enzymes which can convert tricalcium phosphate in the assay medium from insoluble to soluble forms [42, 43]. For example, *B. subtilis* KUPSB16 released 208.18 μg/ml of phosphorus, and there was a correlation between the decrease in pH of the liquid culture medium and the phosphate solubilization rate [44]. In other studies, the decline in pH indicated organic acid production, which suggested that acidification of culture supernatants might be the principal mechanism for phosphate solubilization [45, 46]. Soil bacteria with phosphate solubilization attributes are therefore indispensable in enhancing soil fertility [47]. However, the phosphate solubilization ability may also depend on other factors such as substrate, medium, temperature, time, and other mechanisms in addition to organic acid production [26].

Indole acetic acid is an important phytohormone that regulates plant growth and development [48]. In our study, isolate KK074 was one of the best IAA producers, along with KK077 and KK081. Among the top three IAA-producing isolates, KK074 was uniquely effective in inhibiting the growth of *B. oryzae* 2464. The 16S rRNA gene sequence of KK074 shared high (99.86%) similarity with the genus *Enterobacter*. Bose *et al*. [49] reported that *Enterobacter cloacae* SN19 isolated from rhizosphere of a legume (*Teramnus labialis*) exhibited maximum IAA production (382.23 μg/ml in 0.1% L-tryptophan supplemented medium). Moreover, the IAA-producing *Bacillus methylotrophicus* DD-1, isolated from the root soil of rice plants, was capable of efficiently promoting the growth of rice, and the in vitro production of IAA was recorded as 87.26 μg/ml [50]. However, it has been reported that the levels of IAA produced by bacteria vary across species and it is also influenced by nutrient availability, culture condition, environmental conditions, and substrate preference [51, 52].

In this study, the two most potent bacterial antagonistic isolates were KK024 (*Bacillus* sp.) and KK269 (*Pantoea* sp.) which showed a strong antifungal activity against two fungal pathogens of rice, *C. lunata* NUF001 and *B. oryzae* 2464. Generally, the mechanisms of antagonists on pathogens include nutrition and spatial competition, production of antifungal substances [53], and production of extracellular lytic enzymes [54]. Saechow *et al*. [18] reported that *Bacillus amyloliquefaciens* BAS23 inhibited mycelial growth of *C. lunata* and induced morphological abnormalities, such as hyphal swelling. Moreover, Lee *et al*. [55] and Sharma *et al*. [17] reported that *Bacillus* strains produced antifungal substances that impeded mycelial growth with no physical contact between the bacteria isolates and the pathogen in the inhibition zone.

The cell-free supernatant of KK281 (*Bacillus* sp.) and *Bacillus*-like isolate KK288 displayed the best inhibitory effect on the mycelial growth of *C. lunata* NUF001. It has been reported that many endophytic *Bacillus* spp. secrete various secondary metabolites and cell-free supernatants were tested for their antifungal activity [56]. Cultures of *Bacillus* sp. KK281 were found to produce a wide array of antagonistic substances against pathogenic microorganisms [57]. *Bacillus* species are well known producers of biologically active compounds as lipopeptides with potent antifungal activities [58]. Lipopeptides antifungal substances are frequently found to be secreted by bacteria [59].

The crude extract of KK281, containing surfactin, strongly inhibited growth of *C. lunata* NUF001 and *Xoo*. It has been reported that lipopeptides of the surfactin, iturin and fengycin families have antagonistic activity against a wide range of plant pathogens such as *Fusarium semitectum*, *Helminthosporium oryzae*, *Sclerotinia sclerotiorum* and *Macrophomina phaseolina* [18, 60, 61]. In general, most surfactin biosurfactants belonging to the cyclic lipopeptide family are produced by *Bacillus* spp. [62].

In our study, LC-ESI-MS/MS analysis revealed the presence of surfactin with *m/z* 1036.6947 [M + H]^+^ in crude extract. Surfactin derivative was detected with *m/z* 1036.6947, the mass fragments of 685.4526, 596.4305, 441.2731, and 227.1758. The fragmentation pattern was found to highly match the mass fragments reported by Moro *et al*. [39]. The fragmented mass of 685 is considered as a key mass for the identification of surfactin [63]. In this respect, a similar pattern was reported by Li *et al*. [64] for different surfactin-like homologs in *B. licheniformis* HSN221, when cultivated in MSM medium containing glucose, yeast extract, and ammonium nitrate. Purified surfactin from *Bacillus subtilis* LSFM-05, using raw glycerol as substrate, was shown to have the ESI mass spectra of *m/z* 1022, 1036, 1044, and 1058 [65]. Chen and co-authors also reported surfactin homologs by *B. licheniformis* MB01 at *m/z* 994, 1008, 1022, 1036 with isoforms C12, C13, C14, and C15, respectively [66]. The above analyses revealed that the biosurfactant produced by *Bacillus nealsonii* S2MT strain is a lipopeptide in nature having the highest resemblance with the surfactin family.

In our study, the crude lipopeptide extract was non-cytotoxic towards the L-929 cell line (mouse fibroblast). The cytotoxicity of crude lipopeptides towards cell lines can vary significantly depending on several factors, including the type of lipopeptide, the target cell line, and the concentration of the lipopeptide [62]. In the future, we plan to use purified lipopeptides to better understand the specific effects of lipopeptides on various cell lines.

## Conclusion

This comprehensive study evaluated 315 bacterial isolates in in vitro assay to assess their potential to promote the growth of plants and control diseases. Overall, one isolate (*Bacillus* sp. KK 281) exhibited a strong combination of PGPR characteristics, and antifungal properties against damaging rice pathogens *C. lunata* NUF001 and *X. oryzae* pv. *oryzae*. The lipopeptide extract obtained from isolate KK281 was subjected to advanced LC-ESI-MS/MS analysis, which identified the presence of surfactin. Notably, it was shown that this lipopeptide extract did not demonstrate any cytotoxicity when tested on the L-929 in vitro cell line. As *Bacillus* sp. KK281 has vigorous plant growth-promoting properties, is non-cytotoxic, and has antifungal chemicals, it can now be evaluated in vivo with upland rice in greenhouse and field trials.

## Supplemental Materials

Supplementary data for this paper are available on-line only at http://jmb.or.kr.



## Figures and Tables

**Table 1:** In vitro plant growth promoting properties and antifungal activity.

Bacterial ID Code	IAA production (μg/ml) *	Phosphate solubilization index (PSI) *		pH of medium		Phosphate solubilization (μg/ml) *	Inhibition of pathogenic fungi (%)*	
		3 days	7 days	1 day	7 days		*C. lunata* NUF001	*B. oryzae* 2464
KK007	-	3.44 ± 0.67^bc^	4.74 ± 0.54^b^	7.00	4.06	714.25 ± 12.14^a^	36.59 ± 1.84^cd^	45.06 ± 2.97^bc^
KK024	11.59 ± 1.67^d^	-	-	7.00	4.94	139.12 ± 32.29^f^	58.24 ± 0.82^a^	45.29 ± 2.57^bc^
KK026	16.82 ± 0.60^d^	3.57 ± 1.04^abc^	4.27 ± 0.64^bc^	7.00	5.48	98.17 ± 14.66^g^	-	-
KK066	328.43 ± 24.10^b^	-	2.45 ± 0.15^e^	7.00	5.47	151.52 ± 2.02^f^	30.03 ± 2.41^de^	-
KK067	-	3.48 ± 0.20^bc^	4.82 ± 0.34^b^	7.00	5.81	137.55 ± 1.34^f^	55.02 ± 1.75^ab^	52.83 ± 1.84^ab^
KK070	-	4.09 ± 0.45^ab^	3.49 ± 0.16^d^	7.00	5.32	107.78 ± 3.46^g^	-	-
KK074	362.59 ± 28.02^a^	-	-	-	-	-	-	20.49 ± 8.85^d^
KK075	330.09 ± 15.28^b^	-	-	7.00	4.98	196.75 ± 4.65^e^	0.80 ± 0.15^f^	-
KK077	352.32 ± 13.72^a^	-	-	-	-	-	27.75 ± 0.60^e^	-
KK081	348.71 ± 7.74^ab^	-	-	-	-	-	-	-
KK135	-	-	3.85 ± 0.12^cd^	7.00	3.94	683.45 ± 13.51^b^	-	-
KK138	-	-	2.62 ± 0.03^e^	7.00	3.96	694.63 ± 9.12^b^	-	-
KK184	14.04 ± 2.76^d^	3.87 ± 0.14^abc^	4.71 ± 0.12^b^	7.00	5.56	100.49 ± 2.63^g^	53.23 ± 1.64^ab^	51.47 ± 1.56^ab^
KK225	2.82 ± 0.88^d^	4.51 ± 0.49^a^	7.85 ± 0.54^a^	7.00	4.40	389.55 ± 6.39^c^	38.60 ± 11.97^c^	37.59 ± 8.13^c^
KK269	148.54 ± 1.87^c^	2.98 ± 0.08^c^	3.52 ± 0.14^d^	7.00	5.53	153.02 ± 3.07^f^	50.71 ± 2.68^b^	56.01 ± 3.14^a^
KK275	6.82 ± 0.44^d^	-	-	7.00	4.91	92.11 ± 2.09^g^	54.14 ± 1.34^ab^	47.98 ± 0.47^ab^
KK281	23.59 ± 1.02^d^	-	-	7.00	4.90	275.78 ± 2.87^d^	57.91 ± 0.42^ab^	46.62 ± 5.36^b^

^a-g^The mean values with different superscript letters in a column are statistically different from Duncan’s multiple range test (*p* < 0.05).

**Table 2:** Identification of upland rice endophytic and rhizosphere bacteria based on 16S rRNA gene sequence.

TBRC ID Code	Isolate KK	Sequence Length (bps)	NCBI Accession	Closely related taxa	Similarity (%)	Taxonomic assignment
15994*	KK007	1,466	ON406146	*Pantoea stewartii* subsp. *Indologenes* LMG 2632^T^	99.45	*Pantoea stewartii*
15995*	KK018	1,479	ON406147	*Priestia megaterium* NBRC 15308^T^	99.93	*Priestia megaterium*
15999*	KK066	1,400	ON406148	*Enterobacter roggenkampii* EN-117^T^	99.79	*Enterobacter* sp.
15996^¥^	KK138	1,464	ON406149	*Acinetobacter soli* CIP 110264^T^	100.00	Acinetobacter soli
15997^¥^	KK269	1,468	ON406150	*Pantoea allii* LMG 24248^T^	99.04	*Pantoea* sp.
15998^¥^	KK281	1,473	ON406151	*Bacillus siamensis* KCTC 13613^T^	99.86	*Bacillus* sp.

Note: * = endophytic bacteria, \ = rhizosphere bacteria

**Table 3:** Inhibition zone diameter of lipopeptides extract from isolate KK281 against *Curvularia lunata* NUF001 and *Xanthomonas oryzae* pv. *oryzae* by the paper disc diffusion method.

Sample	Concentration (mg/ml)	Inhibition zone diameter (mm)	
		*C. lunata* NUF001 96 h	*Xoo* 72 h
Crude lipopeptides	12.5	39.93 ± 2.14^a^	19.33 ± 1.29^c^
	6.25	17.20 ± 0.87^c^	16.43 ± 0.50^d^
	3.125	18.10 ± 2.56^c^	12.47 ± 1.23^e^
70% ethanol		0.00 ± 0.00^d^	NA
Prochloraz 500 ppm		29.65 ± 0.77^b^	NA
Streptomycin*		NA	42.50 ± 0.30^b^
Chloramphenicol*		NA	53.20 ± 0.60^a^

^a-e^The mean values with different superscript letters in a column are statistically different from Duncan’s multiple range test (*p* < 0.05).

*Streptomycin 10 μg/disc, chloramphenicol 10 μg/disc
