# Supplementary material for: Screening Plant Growth-Promoting Bacteria with Antimicrobial Properties for Upland Rice
Source: J Microbiol Biotechnol. 2024 Apr 1;34(5):1029–39. doi: 10.4014/jmb.2402.02008 (PMC11180919; doi:10.4014/jmb.2402.02008)
Supplement: Supplementary file 1 [file jmb-34-5-1029-supple.pdf]

Figure S1 **Fig. S1. Inhibition of mycelial growth of fungal pathogens by antagonistic bacteria.** Plates (**A**) control of *C. lunata* NUF001; (**B**) isolate KK024; (**C**) control of *B. oryzae* 2464; (**D**) isolate KK269. Test on PDA plate at 28°C ± 2 for 11 and 21 days, respectively.

Figure S2 **Fig. S2. Inhibitory activity against the growth of *Curvularia lunata* NUF001 by cell-free supernatant of *Bacillus* strains.** (**A**) isolate KK023; (**B**) isolate KK024; (**C**) isolate KK058; (**D**) isolate KK118; (**E**) isolate KK275; (**F**) isolate KK281; (**G**) isolate KK282; (**H**) isolate KK288; (**I**) isolate KK312; (**J**) Prochloraz 500 ppm (control). Test on PDA plate at 28°C ± 2 for 48 hr.

Figure S3 **Fig. S3. Phylogenetic tree using Neighbor-joining approach based on 16S rRNA gene sequences of *Bacillus* isolates.** *Alicyclobacillus acidocaldarius* strain DSM 446^{T} was used as an outgroup. Numbers at nodes indicates levels of bootstrap support (%), based on neighbor-joining analysis 1,000 resampled datasets and the scale bar indicates 0.005 nucleotide substitutions per site.

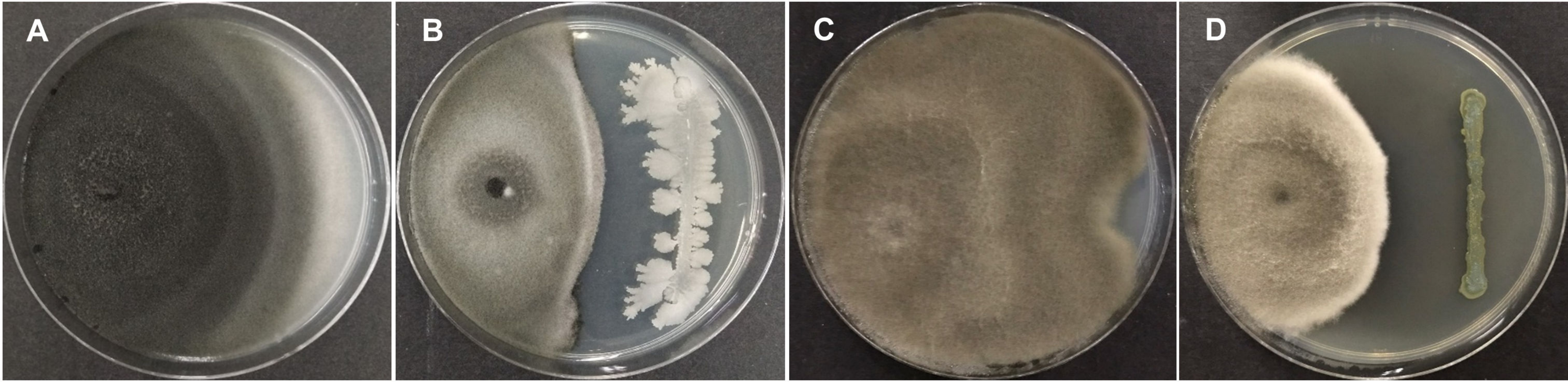

A
B
C
D

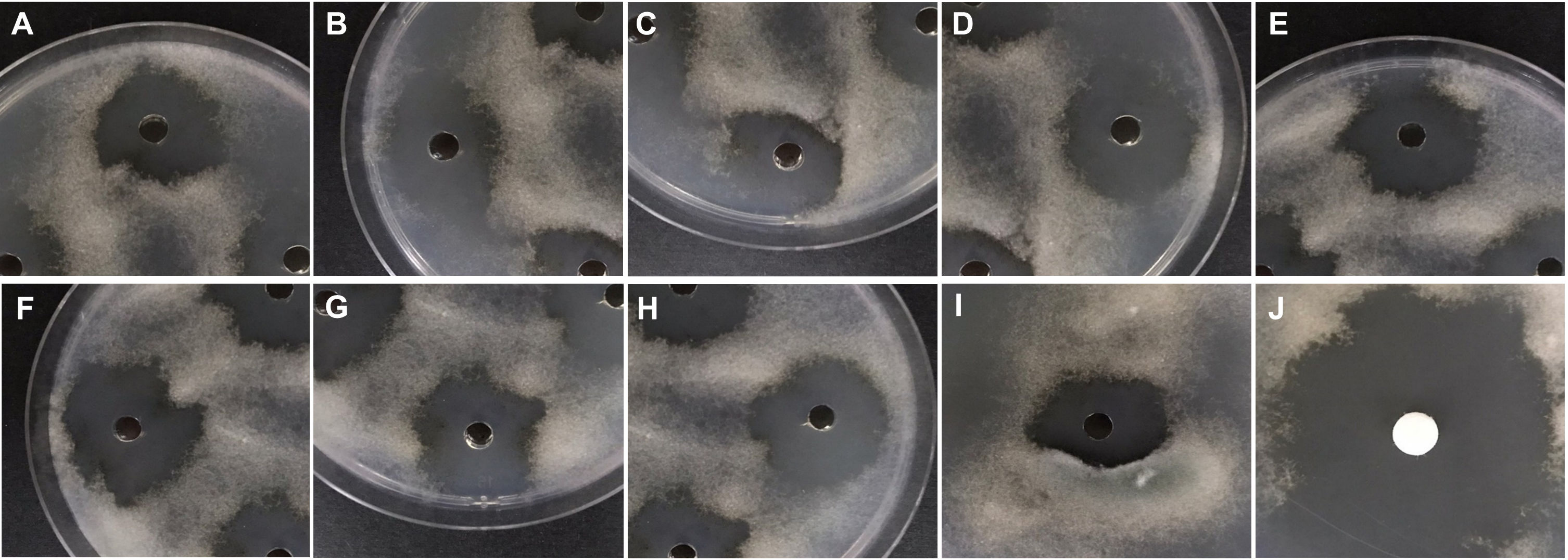
A
B
C
D
E
F
G
H
I
J

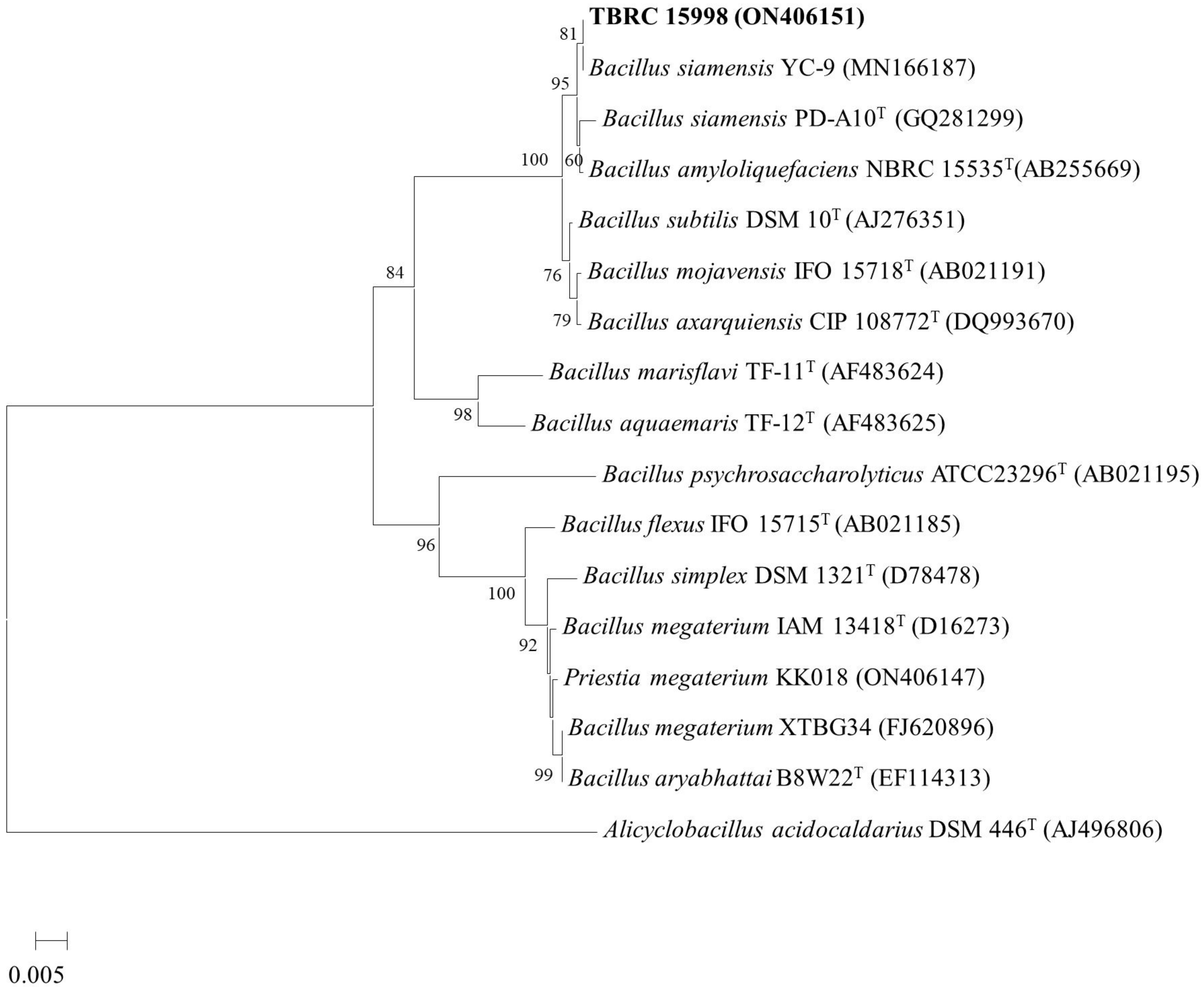

TBRC 15998 (ON406151)
Bacillus siamensis YC-9 (MN166187)
Bacillus siamensis PD-A10T (GQ281299)
Bacillus amyloliquefaciens NBRC 15535T (AB255669)
Bacillus subtilis DSM 10T (AJ276351)
Bacillus mojavensis IFO 15718T (AB021191)
Bacillus axarquiensis CIP 108772T (DQ993670)
Bacillus marisflavi TF-11T (AF483624)
Bacillus aquaemaris TF-12T (AF483625)
Bacillus psychrosaccharolyticus ATCC23296T (AB021195)
Bacillus flexus IFO 15715T (AB021185)
Bacillus simplex DSM 1321T (D78478)
Bacillus megaterium IAM 13418T (D16273)
Priestia megaterium KK018 (ON406147)
Bacillus megaterium XTBG34 (FJ620896)
Bacillus aryabhattai B8W22T (EF114313)
Alicyclobacillus acidocaldarius DSM 446T (AJ496806)
81
95
60
100
76
79
84
98
96
100
92
99
0.005

ภาควิชาชีวเวชศาสตร์ คณะแพทยศาสตร์ มหาวิทยาลัยสงขลานครินทร์
15 ถ.กาญจนวณิชย์ ต.หาดใหญ่ อ.หาดใหญ่ จ.สงขลา 90110
โทรศัพท์ 074-451184 email: siriporn02230@gmail.com

แบบฟอร์มรายงานผลการทดสอบความเป็นพิษต่อเซลล์ (CYTOTOXICITY TEST REPORT)

ชื่อผู้ส่งตัวอย่าง....นางสาวธนัญญา นวลจอน..... ที่อยู่....มหาวิทยาลัยนเรศวร......

โทร.........................083-1588050...... E-mail : ......tananyan62@nu.ac.th........

เลขที่รับงาน (Received no.)...010/2565............ วันที่ขอรับบริการ 21 มีนาคม 2565

Cell type	Sample	IC_{50} (μg/ml)	Remark
L929	TN183	0.029 ± 0.008	ทดสอบที่ความเข้มข้น 0.008, 0.016, 0.032, 0.064, 0.128 μg/ml
	NKS-3	0.054 ± 0.006	
	JA4/5	0.053 ± 0.002	
	KK281	Not inhibited	ทดสอบที่ความเข้มข้น 5, 10, 20, 40, 80 μg/ml

หมายเหตุ

1. Positive Controls : Doxorubicin ค่า IC_{50} เท่ากับ 0.740 μM
2. Not inhibited คือ สารไม่ยับยั้งการเจริญเติบโตของเซลล์ที่ความเข้มข้นที่ทดสอบ 5, 10, 20, 40, 80 μg/ml

ผู้ทดสอบ/Tested by และ ผู้ตรวจสอบ/Checked by

....

(รศ.ดร. พจนพร ไกรดิษฐ์)

อาจารย์ผู้รับผิดชอบ

วันที่ 17 พฤษภาคม 2565

The results are limited to the test condition and further extrapolation is not inferred . Biomedical Science Laboratory will not take any responsibility for any consequences or damages, which may result from this information.

Please note that Biomedical Science Laboratory is not a certification body.

ผลการตรวจสอบนี้เป็นผลจากการทดลองในสภาวะดังกล่าวเท่านั้น ไม่สามารถใช้คาดคะเนผลที่นอกเหนือจากนี้ได้ ห้องปฏิบัติการภาคชีวเวชศาสตร์จะไม่รับผิดชอบในความเสียหายหรือผลใด ๆ ที่เกิดจากข้อมูลนี้ และโปรดทราบว่าห้องปฏิบัติการภาคชีวเวชศาสตร์ไม่ใช่หน่วยงานที่มีอำนาจในการรับรองผลการตรวจสอบใด ๆ ทั้งสิ้น
